# Beta2-Adrenoreceptor Agonists and Long-Term Risk of Parkinson’s Disease

**DOI:** 10.1016/j.parkreldis.2023.105389

**Published:** 2023-03-31

**Authors:** Julia A. Tuominen, Kjetil Bjørnevik, Julia Romanowska, Magne H. Solheim, Thomas B. Grydeland, Marianna Cortese, Clemens R. Scherzer, Trond Riise, Jannicke Igland

**Affiliations:** a Department of Global Public Health and Primary Care, University of Bergen, Årstadveien 17, 5009 Bergen; b Department of Nutrition, Harvard T.H. Chan School of Public Health, 665 Huntington Avenue, Boston, Massachusetts 02115; c Department of Clinical Science, University of Bergen, Jonas Lies veg 87, 5021 Bergen; d Department of Occupational Medicine, Haukeland University Hospital, Bergen, Jonas Lies vei 65, 5021 Bergen; e Neurogenomics Lab, Harvard Medical School, Brigham and Women’s Hospital, 60 Fenwood Road, Boston, Massachusetts 02115; f APDA Center for Advanced Parkinson Research, Harvard Medical School, Brigham and Women’s Hospital, 60 Fenwood Road, Boston, Massachusetts 02115

**Keywords:** Parkinson’s Disease, Beta2-Adrenoreceptor Agonists, Register-based, Nationwide, Drug Prescriptions

## Abstract

**Introduction:**

There is limited information on how the association between Parkinson’s disease and the use of beta2-adrenoreceptor (β2AR) agonists varies among groups of short-, long-, and ultra-long-acting β2AR agonists (SABA, LABA and ultraLABA).

**Methods:**

In this prospective study of the Norwegian population, we estimated the incidence of Parkinson’s disease according to exposure to β2AR agonists as a time-dependent variable by means of Cox regression. We adjusted for educational level, comorbidity and performed a sensitivity analysis excluding individuals with chronic obstructive pulmonary disease (COPD), all factors associated with smoking. Anticholinergics and corticosteroids as drugs with the same indication were analyzed for comparison.

**Results:**

In the follow-up period from 2005 to 2019, 15,807 incident Parkinson’s cases were identified. After adjustments for sex, education and age as the timescale, SABA (Hazard ratio (HR) = 0.84; 95%CI: 0.79, 0.89; p<0.001), LABA (HR = 0.85; 95%CI: 0.81, 0.90; p<0.001) and ultraLABA (HR = 0.6; 95%CI: 0.49, 0.73; p<0.001) were all associated with a lower risk of Parkinson’s disease. After exclusion of COPD patients, corticosteroids and anticholinergics were no longer inversely associated, whereas β2AR agonists remained associated.

**Conclusion:**

Of drugs with the same indication of use, only β2AR agonists remained inversely associated with PD risk after all adjustments, with ultraLABA displaying the overall strongest association. Although the precision of the estimate is limited by the modest number of exposed PD cases without COPD, the association is intriguing and suggest that longer-acting, more lipophilic, and thus likely more brain-penetrant β2AR agonists could be prioritized for further studies.

## Introduction

Several studies have explored the role of β2-adrenoreceptor (β2AR) ligands in Parkinson’s disease (PD). The β2AR is one of the main receptors for norepinephrine, which is released by neuronal projections originating from the locus coeruleus in the brainstem. These norepinephrinergic neurons form arborized axons that project into the substantia nigra, basal ganglia, and throughout the neocortex [1]. The locus coeruleus is damaged during prodromal stages of PD, even preceding nigral dopaminergic neurodegeneration [2].

Mechanistically, selective β2AR activation suppresses degeneration of nigral dopamine neurons, prevents loss of striatal dopamine, and rescues movements in rodent models of PD [3–5]. Complementing the studies on exogenous β2AR-selective ligands, elevation of the synaptic endogenous β2AR ligand, norepinephrine, is also protective [3,6] and this is attenuated by co-treatment with a selective β2AR inhibitor [3]. Molecularly, β2AR agonists downregulate the transcription of the gene SNCA that encode alpha-synuclein [5], a protein linked to autosomal dominant and sporadic PD [7]. β2AR activation also reduces neuroinflammation, another risk factor of PD [4].

The association between a decreased risk of PD and the use of β2AR agonists, which are common therapeutics in obstructive airway diseases, are largely supported by epidemiological evidence, with some discrepant findings [5,8–13]. Yet, data on the effect of different subtypes of β2AR agonists in PD is sparse. As β2AR agonists differ in their onset times and durations of effect, brought about by differences in biochemical properties, they may also be expected to have different effects on mechanisms relevant to PD [14]. Only one of the previous studies included ultra-long-acting β2AR agonists (ultraLABAs) as a separate group, with evidence for a much stronger effect size compared to both short- and long-acting β2AR agonists (SABAs and LABAs) [8]. While this finding is intriguing, data on the effect of ultraLABAs in PD is restricted to the one study in which few were exposed to this subgroup. To our knowledge, none of the clinical or biochemical studies on PD pathology or neuroprotection in general have included ultraLABA in the array of tested substances.

To address this, we conducted a nationwide register-based cohort study to explore the association of different types of β2AR agonists with PD risk using data from the Norwegian Prescription Database (NorPD).

## Methods

### Study population

The study population consisted of all individuals registered as residents in Norway, who possessed a Norwegian personal identification (ID) number, were alive and at least 25 years old as of 1^st^ January 2005 (n=3.2 million), and with no prescriptions for antiparkinsonian drugs in 2004. Information on birth year, sex, emigration year, month, and year of death, as well as level of education were obtained from Statistics Norway (SSB). Data were linked to NorPD to obtain information on prescriptions between the years 2004 and 2019. NorPD was established in 2004 and includes complete information on all prescriptions filled at all pharmacies in Norway, including Anatomical Therapeutic Chemical (ATC) codes, the amount of drug in milliliters or milligrams per prescription, defined daily dose (DDD) indicating the average daily dose per person for the drug’s main indication [15], as well as the associated reimbursement codes. In Norway, drugs for the treatment of certain diseases, such as PD, asthma, and chronic obstructive pulmonary disease (COPD), are covered by the national insurance system, and the reimbursement codes indicate the disease for which the medicine was prescribed. The International Classification of Diseases 10^th^ Revision (ICD-10) is used in specialist care and the International Classification of Primary Care 2^nd^ Edition (ICPC-2) is used in the primary case as the coding system.

To validate PD cases identified based on prescriptions, we used data from the Norwegian Patient Registry (NPR) on outpatient visits and hospitalizations with PD diagnostic codes (ICD-10 G20) during 2010–2019. NPR was established in 2008 and has complete data on all hospitalizations and outpatient visits at somatic and psychiatric hospitals and visits at contracted private specialists from 2009.

The project received ethical approval and exemption from informed consent from the Western Norway Regional Committee for Medical and Health Research Ethics (REK West: 2017/1508). Permissions to use and merge data were obtained from NorPD, NPR and SSB.

### Exposure

Exposure was calculated for all compounds prescribed in Norway in the period 2005–2019 indicated for obstructive airway diseases according to the ATC classification system, with the prefix “R03”. Groups of β2AR agonists (R03AC), anticholinergics (R03BB), and corticosteroids (R03BA) were analyzed separately. β2AR agonists were further grouped into SABA, LABA, and ultraLABA according to their duration of action (Table S1). To account for the use of combination drugs containing either i) β2AR agonists and corticosteroids (R03AK), ii) β2AR agonists and anticholinergics (R03AL), or iii) all three (R03AL08–09, R03AL11–12), these prescriptions were split into their composite parts and analyzed separately using the same date of exposure. For a more detailed explanation, see supplementary table S1. Exposure was defined as a minimum of two prescriptions for a given drug group during follow-up to decrease the possibility of misclassifying drug use. Exposure date was defined as the date of the second prescription and the individual was considered as exposed throughout the remainder of the follow-up time.

### Outcome

To be classified as an incident PD case we required either a minimum of four prescriptions of levodopa (N04BA) together with at least one reimbursement code for PD, or alternatively, a minimum of four prescriptions of monoamine oxidase B (MAO-B) inhibitors (N04BD). The reimbursement code was required in cases that had received levodopa but no MAO-B inhibitors to exclude patients with parkinsonism due to causes other than idiopathic PD. MAO-B inhibitors are very specific to PD as indicated by reimbursement codes and data from NPR. The included reimbursement codes were G20 indicating idiopathic PD (in ICD-10) and 16 indicating parkinsonism according to the coding system used in Norway until 2008.

The date for PD onset was set to the first date of prescription of any antiparkinsonian drug (N04). A total of 15,807 PD cases with onset during 2005–2019 were identified using this algorithm. The identified PD cases were checked against data on hospitalizations and outpatient visits from NPR. Among the 9696 identified cases with PD onset date during 2010–2019, 92% were registered at least once with G20 as primary diagnosis in NPR.

### Analyses

Start of follow-up was set to January 1^st^, 2005. Each person was followed until PD onset, death, emigration, or December 31^st^, 2019, whichever occurred first. Cox regression analysis with drug groups modelled as time-dependent covariates was used to calculate hazard ratios (HR) for PD associated with each drug group. Age was used as the timescale in all models to achieve the best possible adjustment for age.

We tested four models. In Model 1, HR was calculated separately for combined β2AR agonists, anticholinergics, and corticosteroids with adjustment for sex and level of education. An association between smoking and low level of education has been observed in Norway, a health inequality that has become even more pronounced since the 1980s in both men and women [16]. To assess if each drug group was independently associated with PD risk, in Model 2 β2AR agonists, anticholinergics and corticosteroids were mutually adjusted for by being included in the same regression model, in addition to adjustment for sex and education. In Model 3, we assessed confounding by comorbidity and the corresponding medications by including all drug groups at the second ATC level as binary variables, which indicated whether the individual had received at least one prescription from this drug group in the year 2004 prior to the start of follow-up. In this manner we could also adjust for multimorbidity, a combination of adverse health conditions known to be strongly connected to smoking and other lifestyle factors [17].

The role of smoking was further explored in a sensitivity analysis (Model 4) in which potential COPD patients were excluded. The intention was to make those exposed to β2AR agonists and the unexposed more similar with regards to the proportion of smokers. The individual-specific counts of reimbursement codes indicating COPD (ICD-10 J44/J43, ICPC-2 R95, or 45 by the old coding system) and asthma (ICD-10 J45, ICPC-2 R96, or 44 by the old coding system) across all drugs were calculated for each individual. If the indication of use was COPD in 10% or more of the prescriptions, the individual was flagged as a potential COPD patient and excluded from the study population. Models 1, 2, 3, and 4 were repeated for each sub-group of β2AR agonists.

To assess how well we managed to account for smoking by adjusting for education, comorbidity, and by excluding COPD patients, we estimated the relative risk (RR) of nicotine replacement therapy (NRT, N07BA) according to exposure to β2AR agonists. This was conducted using general linear models with log-link and binomial distribution and ever-use of NRT as a binary dependent variable. We compared two separate models: one model with adjustment for age and sex and one model with additional adjustment for educational level, comorbidity, and exclusion of potential COPD patients.

We also calculated E -values that indicate the strength of association that an unmeasured confounder must have with both exposure and outcome to fully account for the observed HR. These were calculated using HRs and associated confidence intervals (CIs) for each drug group after adjusting for sex and age as the timescale and defining the outcome as rare [18].

A dose-response analysis was conducted on β2AR agonists and separately on groups of SABA, LABA, and ultraLABA. This was also done for combined β2AR agonists after exclusion of COPD patients, but not for the three subgroups due to low number of PD cases exposed to the highest doses. A cumulative dose was calculated for each individual and each drug across the exposure period based on the number of daily defined doses (DDDs) specified for each prescription. The 50%, 75%, and 90% quantiles of the distribution of cumulative dose were calculated for each drug group among individuals defined as exposed. The date of reaching 50%, 75%, and 90% quantiles of cumulative DDDs prescribed were found for each individual. Lowest level of use was reached at the date of second prescription, the same threshold as for being defined as exposed. These four levels of drug use were included as time-dependent covariates in a Cox model with age as the time variable and adjustments for sex and level of education. The follow-up time for each individual was split at the date when the cumulative dosage reached the described cut-offs. A test for trend was conducted by including the time-dependent cumulative dose as a continuous variable in the model. Dose-response relationships were also displayed graphically as Kaplan Meier cumulative failure plots with age as the time scale and stratification on category of cumulative dosage [19].

All analyses were performed using R (version 4.1.3) with version 3.2–13 of the survival package [20,21]. Forest plots were created with the forestplot package [22] and dose-response curves with the ggsurvplot function of the survminer package [23]. Significance level was set to 0.05 in all analyses.

### Data Availability

All data were provided by the Norwegian health registries and are available through independent, direct applications to the Regional Committees for Medical and Health Research Ethics (REK) by research teams that wish to utilize the data in their research.

## Results

The characteristics among those unexposed and exposed to β2AR agonists, anticholinergics and corticosteroids are presented in Table 1. The percentage of males exposed to SABA and LABA was somewhat lower than the percentage of males exposed to ultraLABA. Although on average the most common indication of use of β2AR agonists was asthma, the proportion of users with COPD was clearly higher for ultraLABA compared to SABA and LABA. Compared to unexposed individuals, there were notably more individuals in the lowest two educational categories and fewer in the highest educational categories among those exposed to β2AR agonists, corticosteroids and anticholinergics. The difference was most pronounced for the last drug group. Of the three subgroups, the average level of education was slightly lower among those exposed to ultraLABA compared to SABA and LABA.

**Figure 1:**
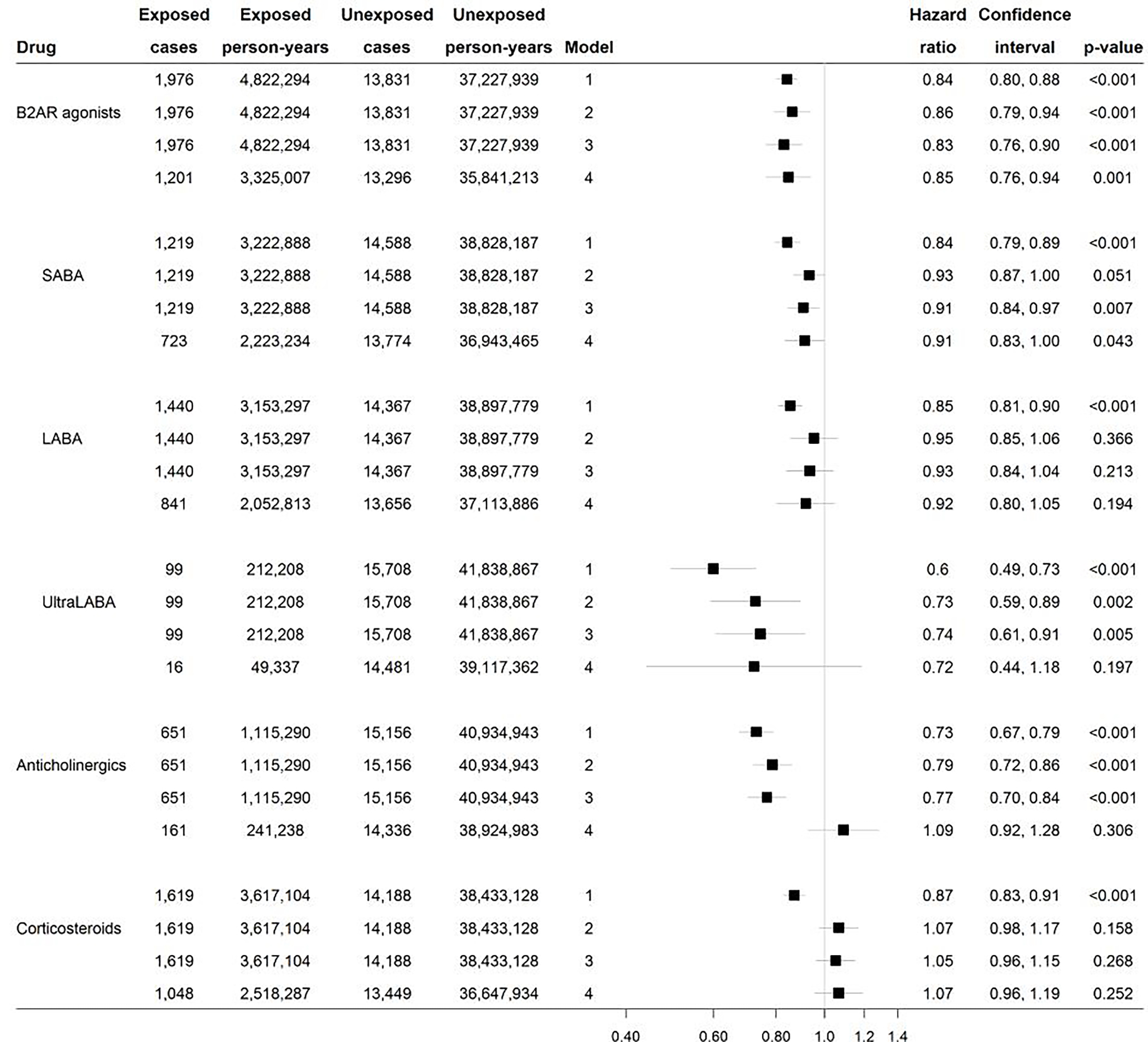
Associations between different drugs for obstructive airway diseases and PD risk. In Model 1, adjustments were made for sex, education, and age as the time variable. In Model 2, additional mutual adjustment was made between β2AR agonists (separately for SABA, LABA, ultraLABA), anticholinergics and corticosteroids. In Model 3, second level ATC codes were added as baseline covariates to adjust for comorbidity. In Model 4, COPD patients were additionally excluded.

All three main drug groups were significantly associated with a reduced risk of PD in a Cox model adjusted for age, sex, and education. Anticholinergics (HR=0.73; 95% CI: 0.67, 0.79; p<0.001) displayed the largest effect size, followed by β2AR agonists (HR=0.84; 95% CI: 0.80, 0.88; p<0.001) and corticosteroids (HR=87; 95% CI: 0.83, 0.91; p<0.001) (Figure 1, Model 1). After mutually adjusting for the three main drug groups, corticosteroids were no longer associated with a reduced PD risk (Figure 1, Model 2). β2AR agonists and anticholinergics remained significantly associated but the estimates were slightly attenuated for β2AR agonists and more so for anticholinergics. When adjusting for comorbidity, the effect estimates were slightly strengthened for each drug group (Figure 1, Model 3).

Each adrenergic subgroup, SABA, LABA, and ultraLABA, was significantly associated with lower PD risk (Figure 1, Model 1). After adjustment for anticholinergics and corticosteroids, only ultraLABA remained significantly associated with PD risk (Figure 1, Model 2). In the model additionally adjusted for comorbidity, the associations of SABA and LABA with PD risk were slightly strengthened, whereas that of ultraLABA remained materially unchanged (Figure 1, Model 3).

After excluding individuals flagged as potential COPD patients, only the group of β2AR agonists (HR=0.85; 95% CI: 0.76, 0.94; p=0.001) retained its association with lower PD risk, whereas neither corticosteroids (HR=1.07; 95% CI: 0.96, 1.19; p=0.252) nor anticholinergics (HR=1.09; 95% CI: 0.92, 1.28; p=0.306) remained associated with PD risk (Figure 1, Model 4). Separate models for each adrenergic subgroup showed a reduced PD risk among those exposed to SABA, which was statistically significant at the 0.05 level. LABA and ultraLABA did not reach statistical significance but retained their negative associations, which remained especially strong for ultraLABA (Figure 1, Model 4).

The RR of using NRT between those exposed and those unexposed to β2AR agonists was reduced from 2.62 (95% CI: 2.59, 2.66; p<.001) to 1.38 (95% CI: 1.36, 1.41; p<.001) after adjusting for educational level, comorbidity, and excluding COPD patients.

E-values for HRs (and the corresponding upper CIs in parentheses) after adjusting for sex and age as the timescale were for combined β2AR agonists 0.60 (0.65), for SABA 0.60 (0.66), for LABA 0.61 (0.68), and for ultraLABA 0.36 (0.47).

**Figure 2:**
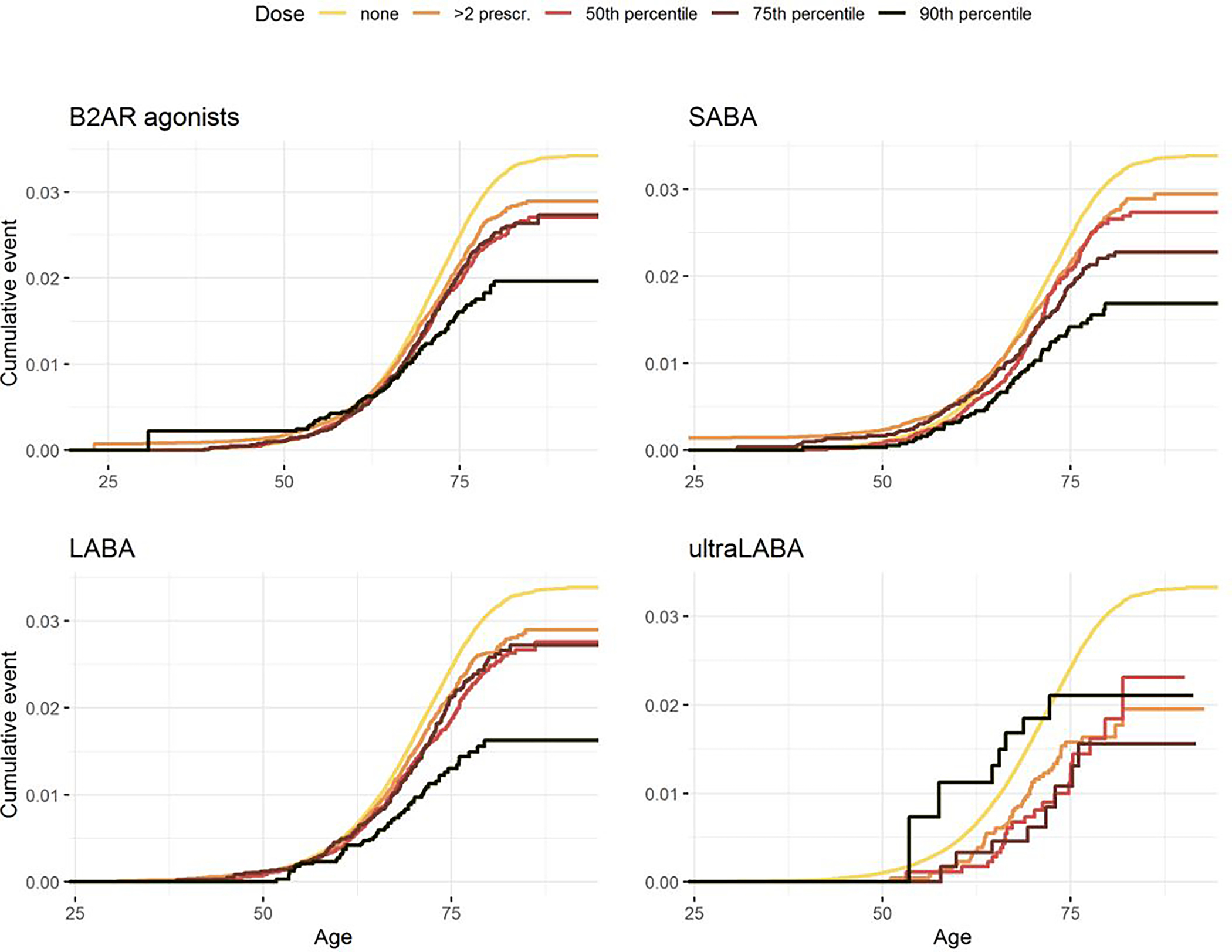
Dose-response relationship between PD risk and β2AR agonists. Cumulative dose is included as a time-varying covariate and calculated based on exposure that exceeds two prescriptions.

In the dose-response analyses, combined β2AR agonists, SABA, LABA, and ultraLABA, all had a significant trend in the relationship with PD risk (Figure 2, Table S2). The association was strongest for the highest dose of each drug in all cases except for ultraLABA, where HR for the highest dose was close to that of the lowest dose, but with considerably wider CI. The test for trend was significant with p-values < 0.001 for β2AR agonists combined and for each subgroup. When excluding COPD patients, the risk of PD was slightly decreased with the lowest dose as well as the 50^th^ and 75^th^ percentiles, while much larger decrease in risk was seen with the highest dose (Figure S1). However, none of the dose levels (Table S3) nor the test of trend (HR = 0.94; 95% CI: 0.89, 1.00; p = 0.055) reached statistical significance.

## Discussion

In this nationwide study, we provide further evidence for a negative association between β2AR agonists and PD. All three subgroups of β2AR agonists remained inversely associated with PD risk in the fully adjusted model and after stratification by indication of use, with ultraLABA displaying the overall strongest association. The observed dose-response relationships are also consistent with a protective effect.

Similar to the present findings, most studies, including a recent meta-analysis of eight cohorts [24], have found the use of β2AR agonists to be associated with lower PD risk [5,8–10,12]. The two studies with null findings assessed exposure to β2AR agonists shortly before disease onset [11,13], which may partly explain why they found no association, as PD has a long prodromal phase [25,26].

Some authors have attributed the association to reverse causation, that persons with prodromal PD symptoms of anxiety and tremor might avoid β2AR agonists due to the potential of these drugs to exacerbate such symptoms [9,11]. We repeated all analyses with a time lag of up to seven years without observing notable changes to the effect estimates (Table S4), thereby finding no support for such alternative explanation.

Some authors reporting an inverse association between β2AR agonists and PD attributed this to confounding by smoking, which is an important source of potential bias [9,12]. β2AR agonists are commonly used for conditions associated with smoking, such as COPD [14], and smoking has consistently been associated with a reduced PD risk [27]. In the only human study that could directly adjust for smoking status, an inverse association was found after adjustment for smoking [8]. Likewise, in a study in which β2AR agonists, anticholinergics, corticosteroids, COPD, and multiple other covariates related to smoking were mutually adjusted for, the inverse association of β2AR agonists with PD risk remained unchanged compared to the model without these adjustments [12,28].

Adjusting for level of education in the present study did not notably influence the risk estimates when compared to a model only controlled for age and sex (data not shown). These results were surprising given the strong association between educational level and smoking in Norway [16], and especially given the markedly decreased risk of PD among individuals with lowest education compared to those with highest education after adjustment for sex and age, with HR 0.67 (95% CI: 0.63, 72; p<0.001). This association is likely driven by smoking, as smoking is, to the best of our knowledge, the only plausible factor that consistently associates positively with low level of education and inversely with PD risk. Therefore, the strong negative association between education and PD observed here is unlikely explained by other factors than smoking. When we compare this risk estimate to the pooled RR of 0.59 between PD and smoking [27], it suggests that educational level is a good indicator of smoking status in our data. Although this does not rule out residual confounding by smoking, it makes it unlikely to fully explain the observed association between β2AR agonists and PD risk.

Unlike COPD, asthma often develops in childhood and is not typically a consequence of long-term smoking [29]. Further, the prevalence of smokers is estimated to be equal among asthma patients and the general population [30,31]. Given these facts, we would expect a diminished association when excluding COPD patients if it were completely explained by smoking. Anticholinergics did not remain associated with PD risk after the exclusion of COPD patients, which suggests confounding by smoking in the association between the drug and PD risk, and that it was successfully controlled for by the exclusion. In contrast, β2AR agonists remained inversely associated with PD risk after the exclusion, which suggests an inverse association beyond that explicable by smoking.

Yet, we found a strong association between the use of β2AR agonists and NRT, which underscores that an association between this drug group and smoking exists in the data. This association was substantially reduced after adjustment for educational level and exclusion of COPD patients, which indicates that these measures taken to reduce confounding by smoking were effective. The odds ratio of attempting smoking cessation for asthmatic smokers versus other smokers have been reported to vary between 1.13 to 2.22 (depending on the strategy) [31]. This may at least partly explain the remaining association between β2AR agonists and NRT. We cannot rule out that some of it is caused by a higher smoking prevalence in the β2AR agonist users compared to non-users, also after removal of COPD patients, but it seems unlikely given that individuals with asthma do not smoke more than the general population [30,31].

E-values give a sense of the strength of association that a confounder would need to have with both the exposure and the outcome to explain an association between the two, which can be useful to evaluate in situations where we lack information on the confounder in the study population. In order to fully explain the association between ultraLABA and reduced PD incidence in this study, the association between smoking and PD would have to be as strong as RR=0.36 [18]. This is much stronger than the pooled RR of 0.59 that has been estimated for the association between smoking and PD risk in the literature [27]. Thus, the strength of association between ultraLABA and PD risk is well above that explicable by confounding through smoking. The effect estimates of combined β2AR agonists, SABA and LABA did not reach a magnitude beyond what is explicable by smoking.

In terms of biological plausibility, long-lasting effects, such as those observed for LABAs and ultraLABAs, are mainly attained through lipophilic properties [32,33]. This is a central factor known to influence the penetrability of a drug through the blood-brain barrier (BBB) [34], suggesting that higher concentrations of longer-acting β2AR agonists reach the central nervous system. Further, due to their chronic administration and longer duration, LABAs, and especially ultraLABAs, may exert a more constant activation of β2AR over time and consequently, a larger effect on PD risk compared to SABAs. Consistent with this possibility and results from a previous study [8], we found ultraLABA to display the largest effect size. However, contrary to findings from previous studies [8,12] that revealed larger effect sizes for LABA compared to SABA, our study did not identify substantial differences in effect sizes between these subgroups. The reason is unclear and may be due to methodological differences, overlapping use of SABA and LABA in the same individuals, or distinct pharmacological properties.

The lack of data on smoking is an important limitation in most such epidemiological studies. In the present study, the presence of residual confounding is possible even after excluding the most likely smokers, that is, COPD patients. The evidence for the role of smoking in asthma etiology is still inconclusive [35–37], which together with the possibility of misclassification of COPD and asthma complicate the interpretation of the present findings. Also, smoking asthmatics have a poorer disease control and an increased demand for medication [38], which gives a reason for caution when interpreting results from the dose-response analyses. However, the disappearing association of anticholinergics after the exclusion of COPD is incompatible with asthma due to smoking driving the results. Another limitation is that PD cases were identified based on filled antiparkinsonian prescriptions and not validated by a treating neurologist. However, we were able to cross-validate our definition of PD cases with diagnostic data, with 92% of the cases identified based on filled antiparkinsonian prescriptions having at least one primary G20 diagnosis in NPR. Further, the number of tests included is high, which elevates the risk of type I error. This should be taken into account when interpreting the results. Due to the strong dependencies among the tested drug groups, three of which (SABA, LABA and ultraLABA) are subgroups of one of them, we did not apply any correction for multiple testing.

The strengths of the present study include a complete population with nationwide prescription data, which secured data from a large population without subsampling and the longitudinal study design. The detailed information available for specific β2AR agonists allowed us to include commonly used combination drugs in the exposure estimations. This provided a better depiction of the association of LABAs and ultraLABAs with PD risk in asthma patients for whom these drugs are not recommended without concomitant use of corticosteroids [14].

### Conclusion

Findings from our study provide further evidence that β2AR agonists are associated with reduced risk of PD, which is unlikely explained solely by smoking due to the following reasons: One would expect attenuation in this association after adjustment for education and comorbidity as factors strongly associated with smoking, and after the exclusion of COPD patients. However, the effect sizes of β2AR agonists remained materially unchanged. Further, anticholinergic users were characterized by lowest educational level and, together with ultraLABA users, had the largest proportion of COPD patients, and therefore also presumably largest proportion of smokers. Yet, anticholinergics were no longer negatively associated with PD risk after all adjustments, whereas β2AR agonists and each subgroup remained associated.

UltraLABA with longest duration of action displayed the markedly strongest association with PD risk. However, the modest number of cases exposed to this drug in a population without COPD makes it difficult to obtain precise estimates from observational data. Whereas SABAs and LABAs have been found to be neuroprotective in rodent models of PD [3–5], the newer ultraLABAs have not yet been mechanistically tested. Given the gap of knowledge concerning the effect of ultraLABAs on PD pathology both at epidemiological and biochemical level, our results warrant further studies to investigate the therapeutic potential of different variants of ultraLABA.

## Supplementary Material

Supplementary material

## Figures and Tables

**Table 1:** Characteristics of individuals unexposed and exposed to β2AR agonists and the three subgroups.

Characteristic	Unexposed 2,600,086^1^	β2AR agonists 570,959^1^	SABA 418,506^1^	LABA 360,467^1^	UltraLABA 68,953^1^	Anticholinergics 187,2331^1^	Corticosteroids 414,6191^1^
Male	1,298,337 (50%)	251,302 (44%)	176,279 (42%)	160,540 (45%)	34,510 (50%)	93,109 (50%)	180,918 (44%)
Diagnosis^2^							
Asthma		337,735 (59%)	254,955 (61%)	231,918 (64%)	31,406 (46%)	66,318 (35%)	280,593 (68%)
COPD		117,437 (21%)	83,186 (20%)	75,843 (21%)	36,762 (53%)	96,652 (52%)	67,425 (16%)
Other^3^		76,983 (13%)	47,108 (11%)	50,488 (14%)	588 (0.9%)	22,814 (12%)	56,898 (14%)
No diagnosis	2,470,414 (95%)	38,804 (6.8%)	33,257 (7.9%)	3,459 (1.0%)	197 (0.3%)	1,449 (0.8%)	9,703 (2.3%)
Education^4^							
primary	50,797 (2.0%)	188,417 (33%)	140,320 (34%)	124,377 (35%)	24,790 (36%)	80,451 (43%)	136,313 (33%)
secondary	667,185 (26%)	254,312 (45%)	184,248 (44%)	160,730 (45%)	32,598 (47%)	82,979 (44%)	184,294 (44%)
undergrad	1,155,509 (44%)	96,303 (17%)	71,088 (17%)	56,378 (16%)	8,729 (13%)	17,425 (9.3%)	70,312 (17%)
graduate	554,155 (21%)	24,005 (4.2%)	16,853 (4.0%)	14,202 (3.9%)	2,101 (3.0%)	4,123 (2.2%)	18,169 (4.4%)
unknown	172,440 (6.6%)	7,922 (1.4%)	5,997 (1.4%)	4,780 (1.3%)	735 (1.1%)	2,255 (1.2%)	5,531 (1.3%)

1 n (%)

2 Defined as the reimbursement code appearing most frequently during the observation period, or the code of the last recorded prescription in case of equal overall frequencies.

3 Such as urticaria, cystic fibrosis, Cushing’s syndrome, allergic asthma.

4 At start of follow-up.
