# Supplementary material for: Beta2-Adrenoreceptor Agonists and Long-Term Risk of Parkinson’s Disease
Source: Parkinsonism Relat Disord. Author manuscript; Available in PMC 2024 May 1. (PMC10387752; doi:10.1016/j.parkreldis.2023.105389)
Supplement: Supplementary material [file NIHMS1914323-supplement-Supplementary_material.docx]

SUPPLEMENTARY MATERIAL

**
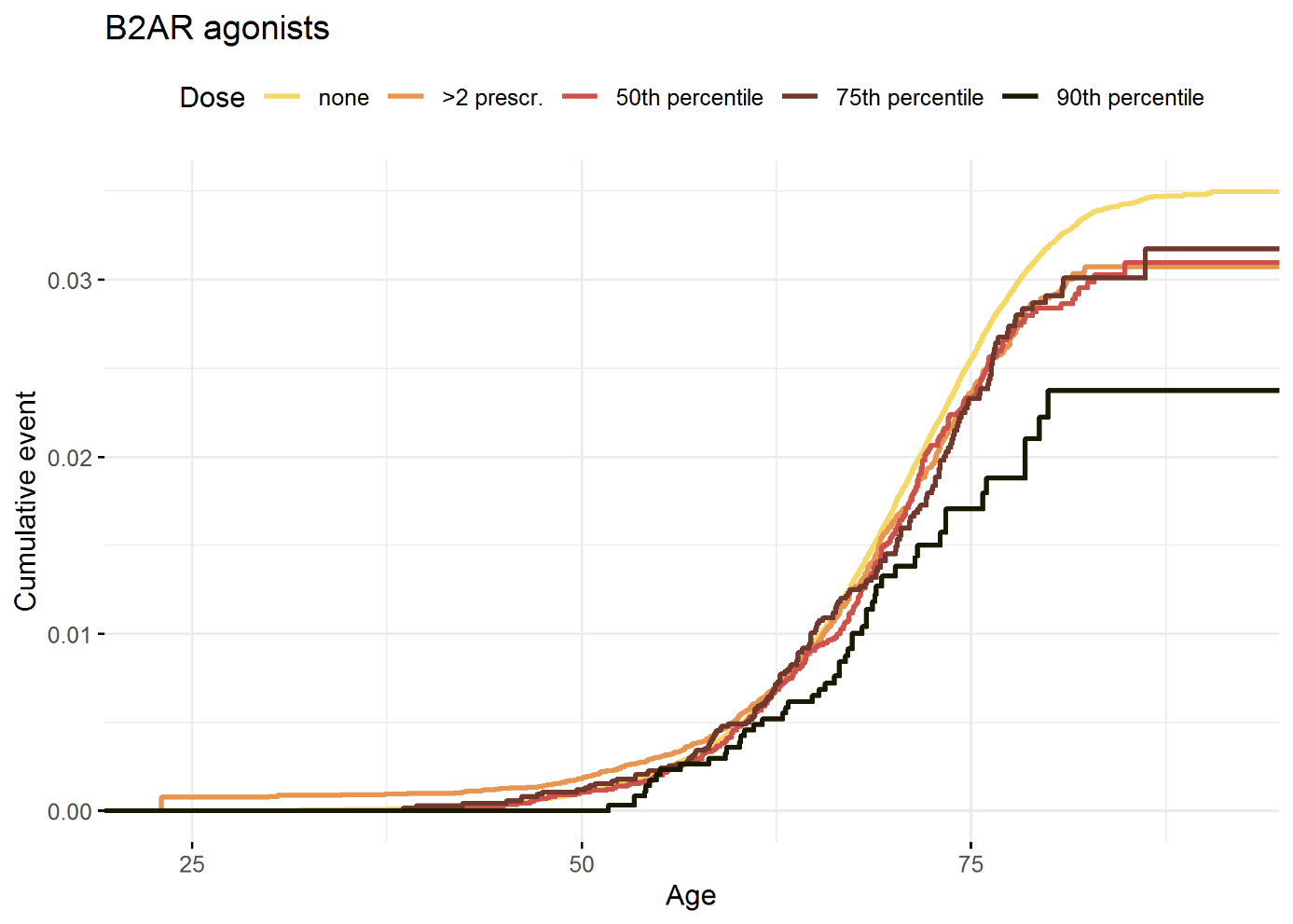
**

***Figure S1.*** Kaplan Meier failure plot according to dose of combined β2AR agonists after exclusion of COPD patients.

| **Table S1** Grouping of β2AR agonists by duration of action | | | |
| --- | --- | --- | --- |
|  | SABA | LABA | ultraLABA |
| ATC (single ingredient) | R03AC02-04 | R04AC12-13 | R03AC18-19 |
| ATC (combined with anticholinergics) | R03AL02 | R03AL05-07 | R03AL03-04 |
| ATC (combined with corticosteroids) | - | R03AK06-08, -11 | R03AK10 |
| ATC (combined with both) |  | R03AL09 | R03AL08 |
| *Note:* For combination products, the amount of each active ingredient per DDD in a selected sample of combination drugs was calculated based on the amount of the active ingredients in one unit dose (UD), multiplied by the number of UDs per DDD (37). The amounts were then compared to those contained in one DDD of the corresponding single-ingredient product. For instance, one DDD of Seretide (R03AK06) contains 100 mcg salmeterol and 100-1000 mcg fluticasone, whereas the respective amounts in single-ingredient products are 100 mcg salmeterol (R03AC12) and 600-1500 mcg fluticasone (R03BA05). The amount of the active ingredients in the products selected for inspection were found to reasonably correspond to those of the single-ingredient products for it to be justifiable to include combination drugs in the calculation of cumulative doses. | | | |

| **Table S2** Dose-response association between β2AR agonists and PD risk. | | | | | | | | | | | |
| --- | --- | --- | --- | --- | --- | --- | --- | --- | --- | --- | --- |
|  | **Adrenergics** | | | | |  | **SABA** | | | | |
| **Dose** | **Person-**  **years** | **Cases** | **HR***^1^* | **95% CI***^1^* | **p-value** |  | **Person-**  **years** | **Cases** | **HR*^1^*** | **95% CI*^1^*** | **p-value** |
| none | 37325799 | 13831 | — | — | — |  | 38929596 | 14588 | — | — | — |
| >2 prescr. | 2787356 | 1019 | 0.89 | 0.83, 0.94 | <0.001 |  | 1907591 | 690 | 0.88 | 0.82, 0.95 | 0.001 |
| 50^th^ percentile | 1161368 | 531 | 0.83 | 0.76, 0.90 | <0.001 |  | 672114 | 283 | 0.88 | 0.79, 0.99 | 0.040 |
| 75^th^ percentile | 608473 | 320 | 0.84 | 0.75, 0.94 | 0.002 |  | 444439 | 180 | 0.77 | 0.67, 0.89 | <0.001 |
| 90^th^ percentile | 275061 | 106 | 0.59 | 0.49, 0.71 | <0.001 |  | 204318 | 66 | 0.58 | 0.46, 0.74 | <0.001 |
|  | | | | | | | | | | | |
|  | **LABA** | | | | |  | **ultraLABA** | | | | |
| **Dose** | **Person-**  **years** | **Cases** | **HR***^1^* | **95% CI***^1^* | **p-value** |  | **Person-**  **years** | **Cases** | **HR*^1^*** | **95% CI*^1^*** | **p-value** |
| none | 38998089 | 14367 | — | — | — |  | 41945638 | 15708 | — | — | — |
| >2 prescr. | 1911557 | 814 | 0.90 | 0.84, 0.97 | 0.005 |  | 135268 | 62 | 0.62 | 0.49, 0.80 | <0.001 |
| 50^th^ percentile | 738236 | 366 | 0.83 | 0.75, 0.92 | <0.001 |  | 45589 | 22 | 0.59 | 0.39, 0.90 | 0.014 |
| 75^th^ percentile | 355455 | 200 | 0.84 | 0.73, 0.97 | 0.016 |  | 19624 | 8 | 0.46 | 0.23, 0.91 | 0.027 |
| 90^th^ percentile | 154721 | 60 | 0.56 | 0.43, 0.72 | <0.001 |  | 11938 | 7 | 0.61 | 0.29, 1.28 | 0.2 |
| *^1^*HR = Hazard Ratio, CI = Confidence Interval  Exposure is drug dosage as a time-dependent covariate and outcome is either onset of PD or censoring, adjusted for age as time scale, sex, and level of education. | | | | | | | | | | | |

| **Table S3** Dose-response association between combined β2AR agonists and PD risk after excluding COPD patients | | | | | |
| --- | --- | --- | --- | --- | --- |
| **Dose** | **Person-years** | **Cases** | **HR***^1^* | **95% CI***^1^* | **p-value** |
| none | 35936074 | 13,296 | — | — |  |
| >2 prescr. | 2115653 | 680 | 0.94 | 0.87, 1.02 | 0.14 |
| 50th percentile | 732830 | 307 | 0.97 | 0.86, 1.08 | 0.6 |
| 75th percentile | 349563 | 165 | 0.97 | 0.83, 1.13 | 0.7 |
| 90th percentile | 133688 | 49 | 0.77 | 0.58, 1.02 | 0.071 |
| *^1^*HR = Hazard Ratio, CI = Confidence Interval | | | | | |

| **Table S4** Time-lag analysis of the association between β2AR agonists and PD risk. | | | | | | | | | | | | | | | |
| --- | --- | --- | --- | --- | --- | --- | --- | --- | --- | --- | --- | --- | --- | --- | --- |
|  | **No lag** | | |  | **2 years** | | |  | **5 years** | | |  | **7 years** | | |
| **Drug** | **HR***^1^* | **95% CI***^1^* | **p-value** |  | **HR***^1^* | **95% CI***^1^* | **p-value** |  | **HR***^1^* | **95% CI***^1^* | **p-value** |  | **HR***^1^* | **95% CI***^1^* | **p-value** |
| β2AR agonists | 0.86 | 0.79, 0.94 | <0.001 |  | 0.90 | 0.82, 0.99 | 0.029 |  | 0.87 | 0.77, 0.98 | 0.019 |  | 0.92 | 0.80, 1.06 | 0.2 |
| SABA | 0.93 | 0.87, 1.00 | 0.051 |  | 0.94 | 0.87, 1.02 | 0.2 |  | 0.92 | 0.83, 1.02 | 0.11 |  | 0.93 | 0.83, 1.05 | 0.3 |
| LABA | 0.95 | 0.85, 1.06 | 0.4 |  | 0.96 | 0.85, 1.09 | 0.6 |  | 0.97 | 0.84, 1.13 | 0.7 |  | 1.07 | 0.90, 1.27 | 0.5 |
| ultraLABA | 0.73 | 0.59, 0.89 | 0.002 |  | 0.77 | 0.58, 1.01 | 0.063 |  | 0.55 | 0.26, 1.16 | 0.12 |  | 0.39 | 0.06, 2.79 | 0.3 |
| *^1^*HR = Hazard Ratio, CI = Confidence Interval  *Note:* Adjusted for age as time scale, sex, level of education, and use of corticosteroids and anticholinergics. Time lag was introduced by requiring the given number of years between exposure and outcome. | | | | | | | | | | | | | | | |
